# Degradation of Multiple Peptides by Microcystin-Degrader *Paucibacter toxinivorans* (2C20)

**DOI:** 10.3390/toxins13040265

**Published:** 2021-04-08

**Authors:** Allan A. Santos, Sylvia Soldatou, Valeria Freitas de Magalhães, Sandra M. F. O. Azevedo, Dolores Camacho-Muñoz, Linda A. Lawton, Christine Edwards

**Affiliations:** 1 Biophysics Institute, Federal University of Rio de Janeiro, 373 Avenida Carlos Chagas Filho, Ilha do Fundão, Rio de Janeiro 21941-901, Brazil; valeria@biof.ufrj.br (V.F.d.M.); sazevedo@biof.ufrj.br (S.M.F.O.A.); 2 School of Pharmacy and Life Sciences, Robert Gordon University, The Sir Ian Wood Building, Garthdee Road, Aberdeen AB10 7GJ, UK; s.soldatou@rgu.ac.uk (S.S.); mariadolores.camacho-munoz@manchester.ac.uk (D.C.-M.); l.lawton@rgu.ac.uk (L.A.L.); c.edwards@rgu.ac.uk (C.E.)

**Keywords:** cyanopeptides, microcystin, anabaenopeptin, aerucyclamide, bacterial degradation rate, UPLC-MS

## Abstract

Since conventional drinking water treatments applied in different countries are inefficient at eliminating potentially toxic cyanobacterial peptides, a number of bacteria have been studied as an alternative to biological filters for the removal of microcystins (MCs). Here, we evaluated the degradation of not only MCs variants (-LR/DM-LR/-RR/-LF/-YR), but also non-MCs peptides (anabaenopeptins A/B, aerucyclamides A/D) by *Paucibacter* *toxinivorans* over 7 days. We also evaluated the degradation rate of MC-LR in a peptide mix, with all peptides tested, and in the presence of *M. aeruginosa* crude extract. Furthermore, biodegradation was assessed for non-cyanobacterial peptides with different chemical structures, such as cyclosporin A, (Glu1)-fibrinopeptide-B, leucine-enkephalin, and oxytocin. When cyanopeptides were individually added, *P. toxinivorans* degraded them (99%) over 7 days, except for MC-LR and -RR, which decreased by about 85 and 90%, respectively. The degradation rate of MC-LR decreased in the peptide mix compared to an individual compound, however, in the presence of the *Microcystis* extract, it was degraded considerably faster (3 days). It was noted that biodegradation rates decreased in the mix for all MCs while non-MCs peptides were immediately degraded. UPLC–QTOF–MS/MS allowed us to identify two linear biodegradation products for MC-LR and MC-YR, and one for MC-LF. Furthermore, *P. toxinivorans* demonstrated complete degradation of non-cyanobacterial peptides, with the exception of oxytocin, where around 50% remained after 7 days. Thus, although *P. toxinivorans* was previously identified as a MC-degrader, it also degrades a wide range of peptides under a range of conditions, which could be optimized as a potential biological tool for water treatment.

## 1. Introduction

The dominance of cyanobacterial blooms causes detrimental effects on aquatic ecosystems, particularly as cyanobacteria release a range of metabolites, many of which may be toxic to different trophic levels [1]. These include microcystins (MCs), the most commonly reported cyanotoxins worldwide with potential human carcinogenic properties [2,3]. MCs are cyclic heptapeptides that include different L-amino acids in two variable positions and chemical variations in all their constituent amino acids, which contribute to the existence of at least 250 variants [3,4]. Among them, MC-LR (containing leucine and arginine) is one of the most toxic to mammals [2,3,4], causing increased liver scores in mice using oral doses from 5 to 10 mg/kg [5]. Additionally, cyanobacteria can produce other peptides that can be potentially toxic, such as aeruginosins, microginins, anabaenopeptins, cyanopeptolins, cyclamides, and microvidirins [6]. Although non-MC cyanopeptides have received less attention than MCs regarding management risks, recent findings suggest this assessment is relevant [7,8,9]. This is supported by the fact that some of these peptides can occur at similar frequency and concentration of MCs in freshwater [6,9] although most studies have been performed qualitatively [6]. Furthermore, some of these peptides exhibit inhibition of proteases, along with ecological effects on species from zooplankton community and accumulation in fish and mussels, although distribution and toxicokinetics are still not completely understood [6,7,10,11,12,13]. Cyanopeptides have been reported in cyanobacterial blooms across the world, with some researchers also reporting the capability of isolated species of *Microcystis* and *Planktothrix* to produce multiple non-MC peptides [14,15].

Regarding their occurrence in the environment, when MCs are released into the water, they can remain relatively stable, regardless of environmental conditions, with half-life values ranging from less than 24 h to 15 days [16,17,18,19]. This fact might be influenced by their initial concentration and microbial community involved in biodegradation [16,19,20,21]. Conversely, there is no clear information about the fate of other cyanopeptides in water systems, since monitoring is difficult due to the lack of standard materials [6,9,10,13].

The exploitation of biodegrading bacteria has been increasing, since they are seen as a safe and low-cost alternative compared to conventional or physical–chemical approaches for the remediation of contaminated water with cyanobacterial compounds [16,21]. Regarding MC biodegradation, many researchers have been contributing to the knowledge of isolated bacteria or microbial communities on the dynamics of this process in different conditions that can be applied as a water treatment process [16,17,18,19,21,22,23]. Although many techniques have been proposed to remove cyanotoxins from water, there are few reports about the application of a permanent and biotechnological system using viable microorganisms as a treatment in situ. This might be due to a limited knowledge of kinetic degradation, interaction with other organisms, or complex compound mixtures, as well as degradation pathway mechanisms [16,21,24].

Previously, there was only one enzymatic pathway clearly described for MC biodegradation, known as the *mlr* pathway, considering biochemical and molecular aspects [23,24,25,26,27]; however, other pathways have been elucidated from different intermediate products or *mlr*-lacking biodegradation activity [19,21,27,28,29,30].

Regarding the *mlr* pathway, it was initially observed in *Sphingomonas* sp. and refers to a gene cluster that generates enzymes responsible for the linearization of the cyclic structure (Mlr A), following a breakdown of linear MC to smaller fragments, such as the tetrapeptide (Adda-Glu-Mdha-Ala) and Adda amino acid by sequential reactions (Mlr B and C) [23,24,25,26,27]. Meanwhile, an enzyme would be involved in the transportation of either entire MCs or their fragments into or out the cells (MlrD) [24,25,27]. Nonetheless, some findings showed that MlrD-lacking strains were still able to degrade MC and excrete the intermediate products out of the cells [31,32]. Recently, researchers have shown that the *paa* gene cluster, responsible for the phenylacetic acid (PAA) degradation pathway, could be closely related to the breakdown of Adda into small fragments, since different genes were significantly upregulated in the presence of MC-LR [33,34]. Thus, using omics analyses, they noticed that homologous enzymes, such as MlrBDAC, PAAase, PaaA, PaaG, and PaaZ were responsible for MC-LR degradation into phenylacetic acid, phenyl-CoA, acetyl-CoA, and then CO_2_ via the classic tricarboxylic cycle [33,34].

In order to compare the capability of *mlr*+ and *mlr*- bacteria to degrade MCs, studies have evaluated some strains in different growth conditions, such as nutrient loading, temperature, and cell density [28,29,35,36], all of which could influence the biodegradation process for water treatment purposes. *Paucibacter toxinivorans* (*mlr*-) had demonstrated the ability to degrade MC-LR in different culture conditions, along with other MCs variants, such as -RR and -YR (R: arginine and Y: tyrosine) [28,29,37]. Despite many studies reporting MC degradation, there are few findings relating to the biodegradation of other cyanobacterial peptides [16,35,38,39,40]. Kato et al. [38], using a cytoplasmic extract of MC-degrading *Sphingosinicella* sp. B-9 strain (*mlr+*), observed enzymes that could also hydrolyze peptide bonds in a range of non-MCs cyclic peptides, suggesting an inherent characteristic to MC-degrading bacteria. Furthermore, Briand et al. [39] showed the degradation of extracellular cyanopeptides, such as aerucyclamides, cyanopeptolins, and MCs (over 3 weeks), by a bacterial community recovered from *Microcystis* mucilage, and added to an axenic *Microcystis* PCC 7806. Recently, Toruńska-Sitarz et al. [40] examined a natural community able to degrade nodularin, a toxic peptide that is similar to MCs. They also observed the biodegradation of peptides belonging to the anabaenopeptin and spumigin families.

Many bacteria with different degradation pathways have been described as specifically for MCs; however, little is known about other potentially toxic peptides. It is therefore of interest to determine the degradation capability of a single bacterium for a wider range of peptides, including those of different chemical structures and biological sources, which could allow it use as a biological tool for the removal of toxic compounds. Therefore, few studies have explored the possible effects of compound interaction on the degradation capability of the respective bacteria.

This present study aims to assess the ability of MC-degrading *P. toxinivorans* to degrade multiple cyanobacterial peptides individually or in mixtures, specifically for the commonly studied MC-LR in a range of conditions, besides other non-cyanobacterial peptides.

## 2. Results

Throughout this study, we investigated the biodegradation of multiple cyanobacterial and non-cyanobacterial peptides by *Paucibacter toxinivorans* (2C20 strain), a strain already known to degrade MC-LR, over 7 days of incubation. Additionally, we carried out an evaluation regarding the biodegradation rate (day^−1^) of all peptides over 7 days of incubation, which was divided into a bi-phasic rate considering different biodegradation rate for the first three days and the last four days (Table S1). We also evaluated the MC-LR biodegradation rate in the presence of different peptides and cyanobacteria crude extract.

### 2.1. Biodegradation of Individual Cyanopeptides

Many of the cyanobacterial peptides were completely degraded by *P. toxinivorans* over 7 days when supplied individually, e.g., DM-LR, MC-LF, MC-YR, ANBP-A, ANBP-B, as well as AC-A and AC-D (Table 1). MC-LR and -RR, however, were not completely degraded during this time, where *P. toxinivorans* reached about 85% and 90% of biodegradation, respectively (Table 1).

**Figure 1:**
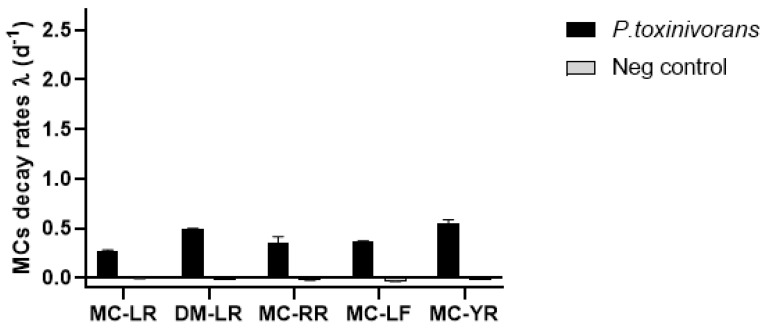
Biodegradation of five microcystins (MCs) variants (-LR, DM-LR, -RR, -LF, and -YR) by *Paucibacter toxinivorans,* showing MCs exponential decay rates over 7 days of incubation with an initial concentration of 10 μg/mL. The data are expressed as mean and standard deviation (*n* = 3).

**Figure 2:**
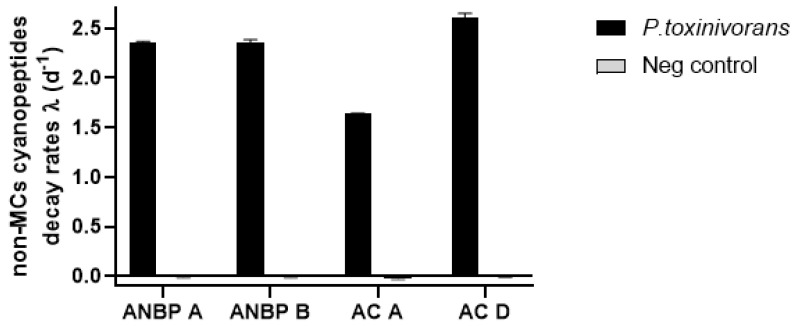
Biodegradation of four non-MC cyanopeptides by *Paucibacter toxinivorans* showing exponential decay rates over the first 3 days of incubation, with an initial concentration of 10 μg/mL. The data are expressed as mean and standard deviation (*n* = 3). ANPB: anabaenopeptin, and AC: aerucyclamide.

Within MC variants, considering the degradation over 7 days, MC-YR showed the highest biodegradation rate (λ = 0.544 ± 0.04; Figure 1, Table S1) with T_1/2_ = 3.5 days while MC-LR had the lowest biodegradation rate (λ = 0.272 ± 0.01; Figure 1, Table S1) with a T_1/2_ of approximately 4.5 days (Table 1). On the other hand, considering a bi-phasic degradation, all MCs were more rapidly degraded in the last 4 days (DM-LR: λ = 0.719 ± 0.003; MC-RR: λ = 0.435 ± 0.095; MC-LF: λ = 0.478 ± 0.012 and λ = 0.819 ± 0.092) than in the first three days (DM-LR: λ = 0.203 ± 0.005; MC-RR: λ = 0.240 ± 0.04; MC-LF: λ = 0.233 ± 0.017 and λ = 0.177 ± 0.023; Table S1). Non-MC peptides were degraded by bacteria at a greater biodegradation rate (d^−1^) than MCs. Meanwhile, all four peptides diminished to below the detection limit after 3 days of incubation with high degradation rate (ANBP-A: λ = 2.363 ± 0.007; ANBP-B: λ = 2.350 ± 0.037; AC-A: λ = 1.643 ± 0.002 and AC-D: λ = 2.607 ± 0.044; Figure 2 and Table S1) where AC-D was the fastest degraded cyanopeptide over this time.

### 2.2. MC-LR Biodegradation in Different Complex Mixtures

**Figure 3:**
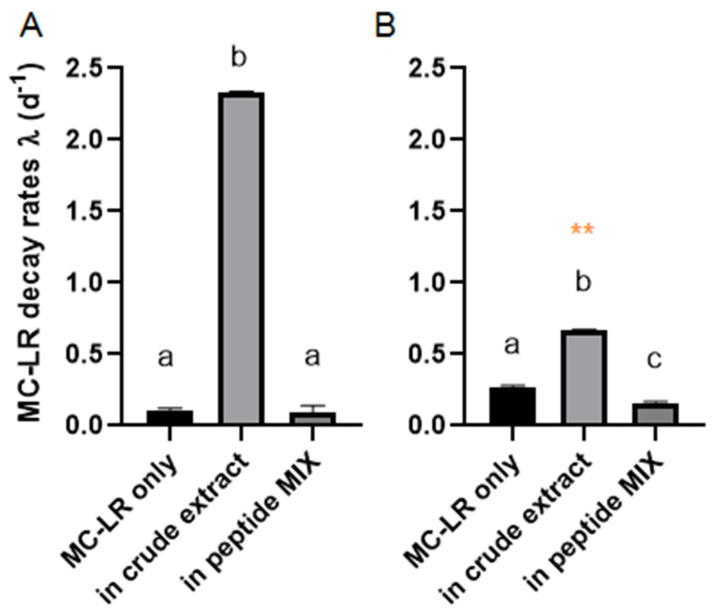
Exponential decay rate λ (d^−1^) of MC-LR by *Paucibacter toxinivorans* in three different conditions: pure MC-LR, in a peptide mix, and in *Microcystis aeruginosa* crude extract over the first 3 days of incubation (**A**), and considering the total 7 days of incubation (**B**). Small letters represent significant different (*p* < 0.05) between the conditions within each chart. Red asterisks mean that the biodegradation rate of MC-LR in *Microcystis* crude extract was extrapolated throughout 7 days, since it was completely degraded in the first 3 days. The data are expressed as mean and standard deviation (*n* = 3).

When MC-LR was the single peptide added to the *P. toxinivorans* liquid culture, there was about 85% of degradation after 7 days of incubation (Table 1) with a respective decay rate of λ = 0.272 ± 0.01 and T_1/2_ = 4.5 days. Moreover, we could observe the degradation rate almost four times higher in the last 4 days (λ = 0.394 ± 0.002) compared to the first three days of incubation (λ = 0.109 ± 0.013). Nonetheless, MC-LR was offered to *P. toxinivorans* at the same concentration (10 μg/mL) into two different conditions: (i) in a peptide mixture consisting of all cyanopeptides previously tested; and (ii) in a *M. aeruginosa* crude extract. The degradation of MC-LR in the peptide mix reached about 60% over the total incubation time (Table 1) and it had a lower decay rate (λ = 0.162 ± 0.008) compared to the condition when it was the only cyanobacterial peptide added (λ = 0.272 ± 0.01) (Figure 3, Table S1). Based on this, the MC-LR half-life in the peptide mix was increased to approximately 5.5 days (Figure 3).

In the presence of *M. aeruginosa* crude extract, *P. toxinivorans* degraded MC-LR at a higher decay rate considering only the first 3 days (λ = 2.33 ± 0.007) (Figure 3 and Table S1). Furthermore, the MC-LR half-life decreased substantially, reaching 1.5 days as opposed to 5.5 days (peptide mix) or 4.5 days (pure peptide). Although MC-LR was completely degraded over 3 days in the crude extract, we can still observe a larger difference in decay rates, extrapolating it throughout the total incubation time (7 days) compared to the biodegradation rate of individual MC-LR and in cyanopeptide mix (Figure 3).

**Figure 4:**
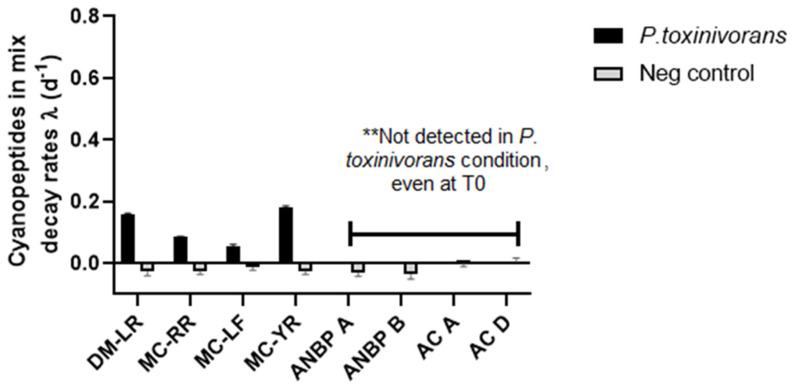
Biodegradation of each cyanobacterial peptide by *Paucibacter toxinivorans* from the mix condition with an initial concentration of 1 µg/mL showing the remaining concentration as exponential decay rates over 7 days of incubation. Non-MCs peptides were not detected even at time 0 in *P. toxinivorans* condition. The data are expressed as mean and standard deviation (*n* = 3). The chart shows four MC variants expressed as DM-LR, -RR, -LF, and -YR besides other cyanopeptides such as ANPB (anabaenopeptin) A and B, and AC (aerucyclamide) A and D.

From the peptide mix condition, we assessed not only MC-LR biodegradation, but also each peptide that was spiked, reaching a final concentration of 1 μg/mL (Figure 4 and Table 1). We observed that not all MCs were completely degraded over 7 days by *P. toxinivorans* with MC-YR showing the highest degradation within MCs (Figure 4 and Table 1). MC-YR and DM-LR ((D-Asp³) MC-LR) both reached about 70% degradation while MC-RR and MC-LF were degraded on average 45% and 35%, respectively (Table 1). The biodegradation rate dropped significantly for all MCs and particularly for MC-LF where the rate decreased of almost seven times (λ = 0.054 ± 0.007). Interestingly, we could not detect any non-MCs peptides (ANBP-A, ANBP-B, AC-A, and AC-D) from *P. toxinivorans* conditions over 7 days, even at time 0. We might consider a very quick biological degradation of all non-MCs cyanopeptides by *P. toxinivorans* since all of them were detected in negative control (Figure S1) where no *P. toxinivorans* was added (Figure 4). The peptide mix had been prepared in a single vial and then added to each well. Moreover, all three microplates were simultaneously set-up; thus, initial sampling times (T0) were taken in an interval of 20–30 min due to the time to prepare the plate. Although it needs further investigation, these findings suggest *P. toxinivorans* is a general cyanopeptide degrader where ANBP-A, ANPB-B, AC-A, and AC-D may be easier to degrade than MCs.

Regarding MC variants, all of them were more rapidly degraded in the last four days compared to the first three days when offered either as individual peptide or in a peptide mix (Table S1), which show a high efficiency of degradation over this interval of time, irrespective of peptides supplied.

### 2.3. Biodegradation of Non-Cyanobacterial Peptides

**Figure 5:**
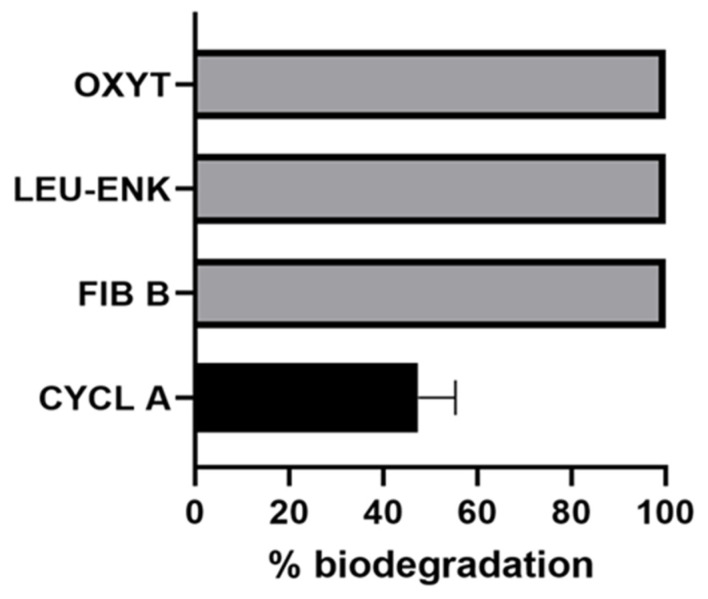
Biodegradation of non-cyanobacterial peptides by *Paucibacter toxinivorans* over 7 days of incubation. The different colors represent complete degradation (grey) or partial degradation (black) over the incubation time. Peptides were abbreviated as oxytocin (OXYT), LEU–ENK (leucine–enkephalin), (Glu1)-fibrinopeptide-B (FIB B), and cyclosporin A (CYCL). The data are expressed in percentage of remaining concentration of peptides, considering the mean and standard deviation (*n* = 3).

Since *P. toxinivorans* successfully degraded all cyanobacterial peptides, we carried out an evaluation of its capability to degrade other peptides from different sources. *P. toxinivorans* was able to completely degrade (Glu1)-fibrinopeptide-B (FIB B), leucine-enkephalin (LEU-ENK), and oxytocin (OXYT) over 7 days of incubation (Figure 5). For cyclosporin A (CYCL), the degradation was about 50% over the course of the incubation (Figure 5).

### 2.4. Identification of MC Biodegradation Products by UPLC–QTOF–MS/MS

MC-LR biodegradation products were identified in the bacterial cultures with pure MC-LR, and in the peptide mix, at days 3 and 7, and they were not present in the control samples. MS^E^ fragmentation of the parent ion *m/z* 1013.5646 corresponding to the degradation product A (4.83 min) revealed a major ion at *m/z* 862.4789 ((M+H-NH_2_-PhCH_2_CHOMe)^+^), corresponding to the linearized MC-LR, which lost the terminal phenylethyl methoxy group and the Adda amino group. MS/MS analysis of the parent ion revealed further fragmentation products of the linearized MC-LR, as shown in Table 2A. An additional linearized biodegradation product of MC-LR with protonated molecular ion at *m/z* 1013.5649 was also identified at retention time 5.6 min. Although the fragmentation pattern of product B revealed ions at *m/z* 488.2812, 304.1642, 175.1196, and 135.0719, which are similar to those of product A and indicative of MCs, ions of higher *m/z,* such as 862, 726, and 571 were not identified (Table 2A, Figure S2). However, an ion at *m/z* 879.5062 was observed and characterized as ((M+H-PhCH2CHOMe)^+^), which corresponds to a linear by-product or MC-LR that has lost the terminal phenylethyl methoxy group. Considering the differences in the mass spectra and retention times of degradation products A and B, we suggest that B might be a structural isomer of A.

Hydrolysis at a different peptide bond is unlikely to have occurred as the daughter ions indicative of the breakage of Arg-Adda bond, such as *m/z* 488.2845, 304.1615, are present in the mass spectra of both linear products. Additionally, analysis of the MS^E^ spectrum of the sample with the peptide mix at day 7 revealed a parent ion at *m/z* 1063.6191 at 4.48 min and 5.16 min, which were assigned as byproducts C and D, respectively, and suggested the presence of linear biodegradation products of MC-YR. For C, the observed major ion at *m/z* 912.4456 was assigned to the linearized MC-YR, which lost the terminal phenylethyl methoxy group and the Adda amino group. MS/MS experiment of the major ion revealed key fragments (Table 2B, Figure S3), which allowed us to assign C as a linear biodegradation product of MC-YR after breakage of at the Arg-Adda bond. For the degradation product D, the major ion at *m/z* 929.4713 indicated the loss of the terminal phenylethyl methoxy group from the MC-YR by-product. The presence of daughter ions at *m/z* 175.1199, 304.1621, and 538.2687 in the MS/MS spectrum of the major ion (*m/z* 929.4713) suggested that product D is an additional linear biodegradation intermediate of MC-YR where the Arg-Adda bond was target by a possible enzymatic mechanism (Table 2B, Figure S3). Moreover, a linear biodegradation product of MC-LF, peptide E, was identified at retention time of 7.65 with a mass-to-charge ratio of 1004.5352 after analysis of the MS^E^ spectrum of the sample with the peptide mix at day 7. MS/MS analysis of the major ion at *m/z* 870.4624 revealed fragmentation patterns, which suggested breakage of the Phe-Adda bond of MC-LF (Table 2C, Figure S4). In specific, the daughter ions at *m/z* 295.1309, 705.3755, 576.3404, and 463.2590 revealed that the linear peptide E was a result of a biodegradation mechanism of MC-LF at the Phe-Adda bond. No biodegradation products were observed for the non-MCs peptides: ANBP-A, ANBP-B, AC-A, and AC-D (Figure S5).

## 3. Discussion

In this current study, we evaluated the capability of bacterium *P. toxinivorans* (2C20 strain) to degrade a range of cyanopeptides, as well as peptides with different chemical structure (cyclic and linear) from eukaryotes. Although *P. toxinivorans* lacks the *mlr* enzymatic pathway, it has been previously described as MC-LR degrader [37] including studies where *P. toxinivorans* 2C20 was used as positive control for the MC-LR biodegradation process [28,29,41]. Recently, authors have suggested bacteria possess other biodegradation pathways than *mlr* from the identifying different patterns of amino acids breaks in degradation-resulting products, besides the absence of *mlr* genes in MC-degrader bacteria [19,21,27,28,29,30,41]. Nevertheless, other findings have shown that a microbe should not necessarily have an exclusive enzymatic pathway to degrade a specific compound, and a single microorganism might be able to degrade a broad range of substances [42,43]. Lezcano et al. [28] observed degradation of MC-LR at 1 mg/L in different nutrient conditions over 5 days, comparing mechanistic pathways from *P. toxinivorans* (*mlr-*), and *mlr+* bacteria, such as *Sphingosinicella microcystinivorans* (Y2 strain) as well as *Sphingopyxis* (from *Sphingomonadales* order). They observed that the Y2 strain completely degraded MC-LR over 10 h in a high total organic carbon and total nitrogen, unlike *P. toxinivorans* that required 120 h to degrade MC-LR in the same conditions, considering a higher lag phase compared to *mlr*+ bacteria. Nonetheless, *P. toxinivorans* was able to completely degrade the toxin over 50 h in low nutrient conditions (i.e., reservoir water and minimal salt medium) when compared to Y2 strain. Later, Morón-Lopez et al. [29] confirmed that *mlr*- bacteria were less efficient in degrading MCs than *mlr*+ regardless of environmental conditions. Moreover, they observed that the addition of nutrients (P and N) and carbon sources, besides a previous exposure to MC, could stimulate *mlr*- bacteria while temperature did not have major influence. In our experiments, Y2 strain did not degrade any cyanobacterial peptides in the three different experimental conditions (individual peptide, peptide mix, and *M. aeruginosa* crude extract) (data not shown). Several factors, such as a long time which bacteria was stored in the fridge prior to performing the experiments, as well as cultivation conditions, may have influenced this finding. These facts could contribute to the loss of its degradation capability, e.g., by inhibiting the expression of *mlr* genes, and that needs further molecular investigations. Meanwhile, *P. toxinivorans* degraded all peptides when added individually. Comparing the degradation of different MC variants by *P. toxinivorans*, Lawton et al. [41] observed a faster biodegradation of MC-LF than MC-LR and MC-RR, which is an observation echoed by our results here. Nonetheless, they found that 50% degradation occurred after 10 days of incubation, slower than our current results, where all individual MCs were effectively degraded over 7 days with a half-life of approximately 4 days, which could be attributed either to MCs initial concentration or to bacterial culture conditions. Our results suggest that MC-LR is degraded slower when compared to other MCs over 7 days, an observation that opposes other studies where it has been suggested that MC-LR degrades more easily compared to MC-RR, -LF, and -YR [35,44]. However, it could be related specifically to *Paucibacter* or other bacteria with alternative pathways than *mlr* and it should not be generalized for all bacteria. Edwards et al. [19] observed different behavior in MC-LR biodegradation by natural microbial communities when only MC-LR was added compared to a mix with three other MCs ((D-Asp^3^)MC-RR, MC-LF, and MC-LW) and nodularin. When the mixture was added to Loch Rescobie water, the half-life of MC-LR increased from 4.5 to 13 days, and it was completely degraded in Forfar Loch water sample over 11 days. When MC-LR was the only peptide added to the Forfar Loch water sample, only 30% degradation occurred in eleven days. Using *Bordetella* sp. strain MC-LTH1, Yang et al. [44] observed a similar biodegradation rate for MC-LR and MC-RR in pure form and in a mixture over 42 h; however, degradation occurred faster when the MCs were added individually (MC-RR: 30 h and MC-LR: 36 h).

Here, MC-LR had a faster decay rate when added as single peptide than in a peptide mix. Regarding the other four MCs in the mix, even at one-tenth lower concentration than individual MC-LR, all were degraded at a lower rate by *P. toxinivorans*. This could be attributed to the structure of MCs, the amount of toxins supplied, or even to a high energetic effort necessary to produce multiple enzymes to act at specific amino acid bonds simultaneously. 

Remarkably, when MC-LR was added into a *M. aeruginosa* crude extract it was completely degraded over 3 days with a half-life of 1.5 days, with the highest decay rate amongst all conditions. Rapala et al. [45] had previously observed a rapid MC biodegradation when crude extracts of the MC-producing *Microcystis* strain were incubated in water or sediment samples taken from a eutrophic lake throughout a cyanobacterial-bloom. According to that study, the bacterial community was able to degrade both MC-LR and (D-Asp^3^)MC-LR within 4 days in bloom-lysate conditions. Similarly, Christoffersen et al. [46] observed a greater degradation rate by a microbial community from a eutrophic lake added to the crude extract of a highly toxic *M. aeruginosa* (Kützing) culture reaching complete removal in 4 days, compared to the extract from a mixed cyanobacterial bloom and *Scenedesmus* sp. culture. The difference could be attributed to the concentration of MCs, since in *M. aeruginosa* culture, it was 18 times more concentrated (54 µg/L) than in the mixed cyanobacterial bloom (3 µg/L), which could increase the degradation capability of the degrading community. In our case, this higher capability to degrade MC-LR in *M. aeruginosa* crude extract than in the cyanopeptide mix could also be explained by the composition of organic compounds from cyanobacterial strain, apart from the peptides individually added, stimulating a faster degradation. Additionally, *M. aeruginosa* can produce many metabolites from multiple peptides beyond MCs [6,9,10,15] to a large set of chemical compounds that remain to be characterized and classified, either as primary or secondary metabolites [47]. This chemical diversity presented in the crude extract might influence the degradation capability of *P. toxinivorans* as well as its growth rate, acting as a synergistic system for degrading specific metabolites, such as MC-LR. This fact was already observed, for example, root exudates in the presence of carbon in soil stimulated bacteria to degrade specific organic compounds [48]. Since *Microcystis* can also produce other MC variants, which, along with other cyanopeptides, decreased the degradation capability of bacteria, this diversity of compounds might also help to avoid a possible toxic effect of them to *Paucibacter*. Unfortunately, we could not explore the composition of crude extract; however, this needs further investigation regarding metabolites interaction for biodegradation purposes. This observation might be interesting for water treatment purposes when metabolite-releasing cyanobacterial blooms require treatment and conventional treatments are not applicable or sufficient.

Irrespective of MC degradation, *P. toxinivorans* completely degraded four other cyanobacterial peptides (AC-A, AC-D, ANBP-A, and ANBP-B) in only 3 days at a higher degradation rate than MCs using the same culture conditions (28 °C, 150 rpm, and initial concentration of 10 μg/mL). ACs are cyclic hexapeptides consisting of three azole/azoline rings likely derived from cysteine and threonine modification [49], whereas ANBPs are cyclic peptides with a characteristic ureido bond connecting the primary amine group with the carboxyl group of the neighboring amino acid to form an amide bond [20,50]. For the first time, we are showing a complete biodegradation of variants within the ANPB family, ANBP-A and B, which Kreitz [35] and Kato et al. [38] observed a very low degradation in their studies. Kreitz [35] evaluated biodegradation using the same strain of *P. toxinivorans* and recorded biodegradation of 4% and 37% for ANPB-A and ANBP-B, respectively. The difference between the Kreitz findings and those of this study could be attributed to the culture maintenance or culture media since nutrient broth was previously used, while in our experiments the R2A broth was used. Using a cytoplasmic extract obtained from MC-degrading *Sphingomonas* bacteria (*mlr* +), Kato et al. [38] also observed degradation of other non-MCs cyanobacterial peptides, such as microviridin, microcyclamide, aeruginopeptin besides MC-LR and attributed it to the possibility of the same degradation pathway of MCs. Nevertheless, we showed that peptides other than MCs, such as ANBP-A, ANPB-B, as well as AC-A and AC-D were quickly degraded by growing non-*mlr* bacteria, which may be easily applied for toxin removal than enzymes from cytoplasmic content. However, we could not verify if bacteria used the same degradation pathway as MCs.

Additionally, these peptides were completely degraded after 3 days with a higher decay rate, compared to MCs. Curiously, in the peptide mix, they may have been degraded within 30 min that corresponded to the time of preparing the experiment and respective sampling. It was noted since they were not detected even at time 0 in *P. toxinivorans* compared to their stability in the negative control over incubation time (Figure S1). Nonetheless, it needs further investigation to evaluate this faster degradation capability compared to MCs with an appropriate experimental design. Through UPLC/PDA coupled to mass spectrometry, several MC degradation products were identified in the culture samples of *P. toxinivorans*. The biodegradation product A, as shown in Table 2, which corresponds to the linearized MC-LR after hydrolysis, was originally characterized by Bourne et al. as a result of enzymatic activity of a *Sphingomonas* species that possess *mlr* pathway [23,24]. Curiously, *P. toxinivorans* (2C20) did not present the *mlr* gene cluster [28,29], although it presented a similar hydrolysis activity to MlrA, cleaving cyclic MC-LR at Arg-Adda bond. Meanwhile, the additional linear peptide B has not been previously reported both in *mlr* and *mlr*-independent pathways [51]. Following several patterns of linear MCs after hydrolysis, as reviewed by Foutiou et al. [51], this fact could suggest an enzyme not yet described from hydrolase family, involved in the biodegradation of MC-LR from a *mlr*-independent *P*. *Toxinivorans*. Although amino acid analysis and chromatographic purification would be required for fully characterizing peptide B, we suggest that this biodegradation product is also formed through hydrolytic cleavage of the Arg-Adda bond in the cyclic MC-LR. Thus, it needs further investigation that can be explored by omics studies.

The biodegradation products of MC-YR follow a similar pattern to those of MC-LR. The identified peptide C corresponds to the linearized MC-YR as a result of enzymatic activity by *P. toxinivorans,* which caused breakage of the Arg-Adda bond. It has been previously reported as an enzymatic biodegradation product of *Sphingopyxis* sp. USTB05 [52]. The biodegradation product D had the same mass-to-charge ratio as C, but their retention times were significantly different, and the fragmentation patterns also differed. However, from the fragmentation pattern, it was concluded that both peptides C and D were formed as a result of the cleavage of the Arg-Adda bond. Similarly, to the hydrolysis of MC-LR, our findings point out linear products from cyclic MC-YR, which were not evidenced by other studies [51], mainly considering *mlr*-independent bacteria. Furthermore, the biodegradation product of MC-LF, named peptide E, was identified through tandem mass spectrometry. To our knowledge, this linearized MC-LF has not been reported before as a result of enzymatic activity from isolated bacteria. Imanishi et al. [53] observed a non-efficient degradation of MC-LF at this structure by isolated *Sphingomonas* B-9 strain (*mlr* +), which they could not assign a similar product with *m/z* 1004.53 to the linear MC by-product.

Here, for the first time, we elucidated linear by-products from different MC variants along with their respective fragmentation pattern after degradation assays using *mlr*-independent bacteria. However, further studies, such as amino acid analysis, purification, and NMR experiments will be required to structurally characterize those by-products. Besides that, *P*. *toxinivorans* should be investigated using omics studies to identify evidences of enzymatic pathways involved in other compounds biodegraded as recently observed for *paa* cluster genes along with *mlr* in MC-LR degradation [33,34].

We also evaluated the ability of *P. toxinivorans* to degrade not only peptides from cyanobacterial organisms, but also from other biological sources and with different chemical structures, such as cyclosporin A (cyclic peptide from eukaryote), (Glu1)-fibrinopeptide-B (linear peptide from human), leucine-enkephalin (linear pentapeptide from eukaryote), and oxytocin (linear peptide vasopressin human hormone). Apart from cyclosporin A, which was ~50% degraded after 7 days, all other three peptides were completely degraded. Cyclosporin A is a cyclic undecapeptide containing a disulfide bond between its Cys residues, and has different amino acids than MCs, such as Tyr-Ile-Gln-Asn [54,55] which also disposes a larger ring structure compared to MCs that can make it more stable and complex to be degraded than cyanopeptides in general. These findings suggest an efficient mechanism of *P. toxinivorans* to degrade a wide range of peptides, from cyanobacterial peptides to human peptides, which shows its large metabolic capability that could be used for remediation purposes. Since many water bodies worldwide are increasingly being polluted with a range of potentially harmful substances, it is extremely important to expand the research into biodegradation and remediation approaches in order to evaluate the bacterial capabilities for degrading a wide range of compounds.

## 4. Conclusions

In this study, we investigated the capability of the already known MC-degrader bacterium *P. toxinivorans* (2C20 strain) to degrade both cyanobacterial and non-cyanobacterial peptides. *P. toxinivorans* demonstrated a high biodegradation rate for all peptides over 7 days of incubation and thereby showed its wide physiological capability to degrade peptides with different chemical structures and sources of origin. Linear by-products of MC-LR, -YR, and -LF, and respective fragmentation patterns were shown for the first time after degradation by *mlr*-independent bacteria. Moreover, *P. toxinivorans* was able to degrade MC-LR at a higher rate in a *M. aeruginosa* PCC 7806 crude extract than purified MC-LR or MC-LR in a peptide mixture. Thus, it could be successfully used for water treatment purposes when there is an MC-producing cyanobacterial bloom and a possible senescence phase as many chemical metabolites, besides peptides, are released into the water body. However, further studies are needed in order to describe the MC pathway by *mlr*-independent *P*. *toxinivorans,* as well as to better understand its biodegradation mechanisms and (non-) toxicity of the produced metabolites that can be released by cyanobacterial species.

## 5. Materials and Methods

### 5.1. Chemicals and Reagents

All microcystins variants (MC-LR, DM-LR, MC-RR, MC-LF, and MC-YR) and other cyanobacterial peptides; anabaenopeptin A and B (ANBP-A and ANBP-B, respectively), aerucyclamide A and D (AC-A and AC-D, respectively) were obtained from Enzo Life Sciences (Exeter, UK). The non-cyanobacterial peptides; cyclosporin-A, (Glu1)-fibrinopeptide-B, leucine-enkephalin, and oxytocin were purchased from Sigma-Aldrich^®^ (Irvine, UK) as well as R2A bacterial culture media, HPLC-grade methanol, acetonitrile, and LC-MS grade formic acid. All peptides and R2A media were solubilized and prepared using ultrapure water at 18.2 MΩ.cm obtained by ELGA^®^ ultrapure water systems (United Kingdom). Peptides were diluted to achieve a final concentration of 10 µg/mL.

### 5.2. Bacterial Cultivation

*Paucibacter toxinivorans* 2C20 strain [37] was obtained from DSMZ-German collection of microorganisms and cell cultures (Leibniz Institute). The strain was streaked on R2A agar medium [56] and incubated at 28 °C over 72 h. A single colony from the agar plate was initially transferred to 20 mL of R2A broth in a shaker incubator (150 rpm) over 24 h at 28 °C. Then, 1 mL was transferred to 100 mL of R2A broth and incubated at the same conditions over 72 h in order to obtain the growth curve measured by optical density at 600 nm (OD600) and to identify the mid-log phase, which point, an active stage of metabolism and bacterial growth. To prepare all experiments, *P. toxinivorans* was grown as subcultures in the same media (R2A media, 1:100 diluted in 100 mL) and conditions (28 °C, 150 rpm) over 24 h until it reached mid-log phase where OD was corrected to 0.4 with phosphate-buffer saline (PBS) before starting the experiment.

### 5.3. High-Throughput 96-Well Microplate Biodegradation Experiments

OD-corrected *P. toxinivorans* was transferred to the 96-well microplate in a solution with fresh culture media and individual peptides. For all experimental conditions, microplates were prepared to allow different sampling times over 7 days of incubation in a shaker incubator at 150 rpm and 28 °C. Three different plates were initially prepared maintaining the same conditions, and they were, respectively, taken to be considered as sampling time at 0, 3, and 7th days. This procedure was established in order to sample only once from each well to avoid the effect of differing volumes and potential contamination. At each sampling time, 150 µL were taken and added to 150 µL of 80% aqueous methanol solution with 2% formic acid (in a 1.5 mL centrifuge tube) in order to kill bacteria and stop their metabolism, as well as to precipitate large proteins, which could affect analytical procedures. Samples were centrifuged for 15 min at 13,000× *g*. Supernatants were recovered, 1:10 diluted (100:900 μL) in aqueous methanol 80% and stored at −20 °C until ultrahigh performance liquid chromatography coupled to mass spectrometry analysis (UPLC–MS). This experimental design was applied in all experiments in order to evaluate the biodegradation of peptides by *P. toxinivorans*. All experimental conditions were carried out in biological replicates (*n* = 3) and with negative controls without bacteria.

#### 5.3.1. Biodegradation of Individual Cyanopeptides

The first experimental condition consisted of eight single cyanobacterial peptides (DM-LR, MC-RR, MC-LF, MC-YR, ANBP-A, ANBP-B, AC-A, and AC-D) added individually at a final concentration of 10 μg/mL for each one. The final volume of 150 µL was composed of 5 µL OD-corrected bacterial culture, 5 µL individual peptide, and 140 µL sterile R2A media. Samples were taken at 0, 3, and 7 days of incubation.

#### 5.3.2. Biodegradation Rate of MC-LR in Complex Mixtures

To compare MC-LR biodegradation rate by *P. toxinivorans* in a more complex chemical mixture, 10 μg/mL of this variant (in 5 µL) was added to OD-corrected bacteria (5 µL) in a mixture of cyanopeptides and in a *M. aeruginosa* crude extract diluted in R2A media (140 µL). The cyanopeptide mix consisted of all cyanopeptides previously tested (DM-LR, MC-RR, MC-LF, MC-YR, ANBP-A, ANBP-B, AC-A, and AC-D), which was prepared in R2A media (140 µL) to achieve a final concentration of 1 µg/mL. *M. aeruginosa* (PCC 7806) crude extract was obtained from a supernatant produced by freezing–thawing (−20 °C) and centrifuged over 15 min at 13,000 × *g*. The amount of MC-LR present in the resulting supernatant of the crude extract was quantified through UPLC–PDA–MS at 2.45 µg/mL. Then, it was diluted 1:10 in R2A media and 140 µL were added within each well with bacteria (5 µL) and 10 µg/mL MC-LR (5 µL). All conditions were as before (Section 5.3) with samples taken at 0, 3, and 7 days of incubation.

#### 5.3.3. Biodegradation of Non-Cyanobacterial Peptides

To investigate the biodegradation of four single non-cyanobacterial peptides with different chemical structures and biological sources (cyclosporin A: cyclic peptide from fungus *Tolypocladium inflatum*; (Glu1)-fibrinopeptide-B: linear peptide from human; leucine-enkephalin: linear peptide from mammalian brain; oxytocin: cyclic peptide mammalian hormone) in final concentrations of 10 μg/mL. The final volume of 150 µL in each well was composed of 5 µL OD-corrected bacteria, 5 µL purified peptide, and 140 µL sterile R2A media. Samples were taken at 0 and 7 days of incubation. 

### 5.4. Analysis of Peptides by Ultrahigh Performance Liquid Chromatography Coupled to Photodiode Array Detection and Tandem Mass Spectrometry (UPLC–PDA–MS/MS)

All peptides studied were analyzed by UPLC–PDA–MS/MS (Waters^®^, Manchester, UK) as described previously by Turner et al. [57]. Briefly, chromatographic separation was carried out using a Waters ACQUITY UPLC BEH C18 column (1.7 µm, 2.1 × 100 mm) held at 60 °C. Samples were kept in the sample manager at 10 °C and the injection volume was 5 µL. Mobile phase consisted of (A) water + 0.025% formic acid and (B) acetonitrile + 0.025% formic acid at a flow rate of 0.6 mL/min. The gradient consisted of 2% B initial condition rising to 25% B at 0.5 min holding until 1.5 min, rising to 40% B at 3.0 min, increasing further to 50% B at 4 min, a quick rise to 95% B and 4.1 min and held until 4.5 min before dropping back to 2% B at 5 min. Detection wavelength was set at 238 nm. Mass spectrometric detection was performed with a triple quadrupole mass spectrometer (Waters Xevo TQ-XS, Manchester, UK) equipped with an electrospray ionization (ESI) interface operating in positive ionization mode. Operational parameters were as follow: 150 °C source temperature, 600 °C desolvation temperature, 600 L/h desolvation gas flow (N_2_), 150 L/h cone gas flow, and 0.15 mL/min collision gas flow (Ar). Capillary voltage was held at 1.0 kV. Multiple Reaction Monitoring (MRM) transitions used as well as retention time, chemical structure, and respective formula of peptides can be found in Table S2. Quantification was performed using external calibration curves based on six-point calibration for each peptide analogue ranging from 0.5 to 500 ng/mL. The detection limit was 0.5 ng/mL and quantification limit 1.0 ng/mL. Acquisition and processing of MS data were done using MassLynx v 4.2 software (Waters^®^, Manchester, UK).

### 5.5. Analysis of Peptide Biodegradation Products by UPLC–PDA Coupled to Quadrupole Time of Flight Mass Spectrometry (QTOF-MS^E^, QTOF-MS/MS)

Analysis of the MCs biodegradation products were analyzed using UPLC–PDA–QTOF–MS^E^ and –MS/MS (Waters, UK) equipped with an ESI source. Separation was carried out on a C18 BEH column (1.7μm, 100 × 2.1 mm) held at 40 °C. Mobile phase was acetonitrile with 0.1% formic acid (B) and water with 0.1% formic acid (A) at a flow rate of 0.2 mL/min. Gradient elution was as follows: 20% B initial conditions rising to 70% B at 9.50 min, increasing further to 100% B at 10 min, holding until 11 min, dropping back to 20% B at 12 min and holding until 14 min. The QTOF was operated in positive ESI mode. Parameters were 3.0 kV capillary voltage, 40 V cone voltage, 100 °C source temperature, 250 °C desolvation temperature, 150 L/h cone gas flow, 600 L/h desolvation gas flow. Argon was used as the collision gas. MS/MS consisted of four functions: the first function used collision energy ramp of 20–50 eV to acquire MS^E^ data, the second and third function used a collision energy ramp of 20–50 eV for the targeted masses at a scan time of 0.1 s and the fourth function acquired the lock mass data for online mass calibration. Leucine–Enkephalin (*m/z* 556.2771 for positive electrospray mode) was infused at a flow rate of 10 μL/min at 10 s intervals as lock mass. Acquisition and processing of MS data were done using MassLynx v 4.2 software (Waters^®^, Manchester, UK).

### 5.6. Biodegradation Rate, Half-Life, and Statistical Analysis

Biodegradation of cyanobacterial peptides was also determined from an exponential decay rate, the formula for which is represented by Cy(t) = Cy_0_ × e^−λt^, where Cy(t) is the respective cyanopeptide concentration (μg/mL) at time t, Cy_0_ is the concentration at day 0, and λ is the decay rate as decimal value (day^−1^). In order to evaluate different responses besides degradation rate over 7 days of incubation, two calculations were made: over the first 3 days and over the last 4 days of incubation. Moreover, we also considered the half-life (T_1/2_) for each cyanobacterial peptide when it reached approximately 50% of degradation according to biodegradation charts. To compare the MC-LR biodegradation rate under different conditions such as: individual one, MC-LR in the peptide mix, and MC-LR in the *Microcystis* crude extract, a one-way ANOVA was applied to test the null hypothesis that the biodegradation rate between these conditions was the same; while the alternative hypothesis considered them to be different (*p* < 0.05). All statistical analyses were carried out on GraphPad Prism 8.4.

## Figures and Tables

**Table 1:** Remaining cyanopeptide concentration at each sampling time of *Paucibacter toxinivorans* (2C20) degradation. Five MC variants (MC-LR, DM-LR, MC-RR, MC-LF, and MC-YR) as well as four non-MC cyanopeptides (anabaenopeptins: ANPB-A and -B; aerucyclamide: AC-A and -D) were evaluated in different experimental conditions. The data are represented as mean ± standard deviation (*n* = 3). nd means cyanopeptides were not detected at the respective time (either 3 or 7th day) and asterisk means cyanopeptides were not detected even at T0.

Peptide	Peptide Remaining Concentration (%)			
	*Paucibacter toxinivorans*		Negative Control	
	3rd Day	7th Day	3rd Day	7th Day
**Individual**				
MC-LR	72 ± 2.8	15 ± 1.1	104 ± 2.8	106 ± 3.4
DM-LR	54 ± 0.8	0.7 ± 1.3	106 ± 2.2	119 ± 0.3
MC-RR	49 ± 6.1	9 ± 4.1	101 ± 8.2	119 ± 4.4
MC-LF	49 ± 2.5	nd	143 ± 4.5	126 ± 5.5
MC-YR	59 ± 4.0	1 ± 1.7	115 ± 7.5	119 ± 0.4
ANBP-A	nd	nd	112 ± 8.1	112 ± 10.1
ANBP-B	nd	nd	100 ± 17.1	99 ± 18.1
AC-A	nd	nd	125 ± 14.1	126 ± 14.3
AC-D	nd	nd	113 ± 1.6	102 ± 4.6
**In MIX**				
MC-LR	77 ± 11.6	32 ± 1.9	112 ± 6.8	121 ± 11.9
DM-LR	77 ± 5.4	33 ± 1.1	108 ± 5.2	121 ± 11.3
MC-RR	81 ± 5.9	54 ± 0.7	109 ± 6.2	119 ± 9.9
MC-LF	94 ± 7.2	68 ± 3.2	107 ± 3.4	109 ± 8.2
MC-YR	72 ± 4.3	28 ± 0.7	107 ± 5.1	118 ± 10.3
ANBP-A	nd *	nd *	111 ± 7.7	123 ± 11.1
ANBP-B	nd *	nd *	116 ± 9.5	129 ± 4.5
AC-A	nd *	nd *	109 ± 5.6	101 ± 7.6
AC-D	nd *	nd *	103 ± 6.9	97 ± 8.2
**In crude extract of *M. aeruginosa***				
MC-LR	0.9 ± 0.02	0.9 ± 0.02	100 ± 3.9	104 ± 7.6

**Table 2:** Parent and daughter ions from linearized MCs biodegradation products, considering products A and B for MC-LR in (**A**), products C and D for MC-YR in (**B**), and product E for MC-LF in (**C**).

**(** **A)**			
**MC-LR Linear By-Product A**		**MC-LR Linear By-Product B**	
***m/z***	**Identified Structure**	***m/z***	**Identified Structure**
1013.5646	M+H, Adda-Glu-Mdha-Ala-Leu-Masp-Arg-OH+H	1013.5649	M+H, Adda-Glu-Mdha-Ala-Leu-Masp-Arg-OH+H
862.4684	M+H-NH_2_-PhCH_2_CHOMe	879.5062	M+H-PhCH_2_CHOMe
756.3956	CH_3_-CHCO-Glu-Mdha-Ala-Leu-Masp-Arg-OH+2H	821.4431	Not detected
726.3453	CO-Glu-Mdha-Ala-Leu-Masp-Arg-OH+2H	743.3694	Not detected
-	-	663.3569	Not detected
571.3195	Mdha-Ala-Leu-Masp-Arg-OH+2H	588.3467	Not detected
488.2766	Ala-Leu-Masp-Arg-OH+2H	488.2812	Ala-Leu-Masp-Arg-OH+2H
375.1940	C_11_H_14_O-Glu-Mdha+H	363.1936	Not detected
304.1622	Masp-Arg-OH+2H	304.1642	Masp-Arg-OH+2H
286.1524	Masp-Arg+H	286.1516	Masp-Arg+H
175.1189	Arg-OH+2H	175.1196	Arg-OH+2H
135.0814	PhCH_2_CHOMe	135.0719	PhCH_2_CHOMe
**(B)**			
**MC-YR Linear By-Product C**		**MC-YR Linear By-Product D**	
***m/z***	**Identified Structure**	***m/z***	**Identified Structure**
1063.6191	M+H, Adda-Glu-Mdha-Ala-Tyr-Masp-Arg-OH+H	1063.6200	M+H, Adda-Glu-Mdha-Ala-Tyr-Masp-Arg-OH+H
912.4456	M+H-NH_2_-PhCH_2_CHOMe	929.4713	M+H-PhCH_2_CHOMe
-	-	893.4518	Not detected
621.3013	Mdha-Ala-Tyr-Masp-Arg-OH+2H	731.3482	Not detected
-	-	638.3301	Not detected
538.2344	Ala-Tyr-Masp-Arg-OH+2H	538.2687	Ala-Tyr-Masp-Arg-OH+2H
450.2138	Not detected	-	-
375.1960	C_11_H_14_O-Glu-Mdha+H	363.1893	Not detected
304.1624	Masp-Arg-OH+2H	304.1621	Masp-Arg-OH+2H
286.1526	Masp-Arg+H	286.1523	Masp-Arg+H
213.1116	Glu-Mdha+H	-	-
175.1196	Arg-OH+2H	175.1199	Arg-OH+2H
**(C)**			
**MC-LF Linear By-Product E**			
***m/z***	**Identified Structure**		
1004.5352	M+H, Adda-Glu-Mdha-Ala-Leu-Masp-Phe-OH+H		
870.4624	M+H-PhCH_2_CHOMe		
853.4357	M+H-NH_2_-PhCH_2_CHOMe, (M+H-151)		
705.3755	(Masp-Leu-Ala-Mhda-Glu-Adda-OH-H)-151		
576.3404	(Leu-Ala-Mhda-Glu-Adda-OH-H)-151		
463.2590	(Ala-Mhda-Glu-Adda-OH-H)-151		
435.2520	Not detected		
418.2407	Not detected		
375.1956	C_11_H_14_O-Glu-Mdha+H		
363.1892	Not detected		
335.1969	Not detected		
295.1309	Masp-Phe-OH+2H		
206.1505	Not detected		
163.1154	PhCH_2_-CHOCH_3_CHCH_3_		
135.1164	PhCH_2_CHOMe		

## Data Availability

Data are contained within the article or supplementary materials.

## Supplementary Materials

The following are available online at https://www.mdpi.com/article/10.3390/toxins13040265/s1, Figure S1: Negative control without *Paucibacter toxinivorans* culture for cyanopeptides degradation into mix condition. The data are expressed in percentage of remained concentration of peptides considering mean and standard deviation (*n* = 3), Figure S2: (A) Bottom: MS^E^ spectrum of biodegradation product A at 4.83 min identified in the *Paucibacter toxinivorans* 2C20 culture sample in pure MC-LR conditions at day 7, Top: MS^E^ spectrum of biodegradation product B at 5.60 min identified in the *Paucibacter toxinivorans* 2C20 culture sample in in pure MC-LR conditions at day 7. (B) Bottom: MS/MS spectrum of *m/z* 862.4789, Top: MS/MS spectrum of *m/z* 879.5062, Figure S3: (A) Bottom: MS^E^ spectrum of biodegradation product C at 4.48 min identified in the *Paucibacter toxinivorans* 2C20 culture sample in peptide mixture condition at day 7, Top: MS^E^ spectrum of biodegradation product C at 5.16 min identified in the *Paucibacter toxinivorans* 2C20 culture sample in peptide mixture condition at day 7. (B) Bottom: MS/MS spectrum of *m/z* 912.4462, Top: MS/MS spectrum of *m/z* 929.4723, Figure S4: (A) MS^E^ spectrum of biodegradation product E at 7.66 min identified in the *Paucibacter toxinivorans* 2C20 culture sample in peptide mixture condition at day 7, (B) MS/MS spectrum of *m/z* 870.4624., Figure S5: (A) UPLC chromatogram of the control (top) and of the *Paucibacter toxinivorans* 2C20 culture at day 3 (bottom), AC-A at 5.38 min (B) MS^E^ spectrum of AC-A in the control (top) and in the *Paucibacter toxinivorans* 2C20 culture at day 3 (bottom). (C) UPLC chromatogram of the control (top) and of the *Paucibacter toxinivorans* 2C20 culture at day 3 (bottom), AC-D at 4.76 min (D) MS^E^ spectrum of AC-D in the control (top) and in the *Paucibacter toxinivorans* 2C20 culture at day 3 (bottom). (E) UPLC chromatogram of the control (top) and of the *Paucibacter toxinivorans* 2C20 culture at day 3 (bottom), ANBP-A at 4.89 min (F) MS^E^ spectrum of ANBP-A in the control at day 3. No ANBP-A was observed in in the *Paucibacter toxinivorans* 2C20 culture at day 3. (G) UPLC chromatogram of the control (top) and of the *Paucibacter toxinivorans* 2C20 culture at day 3 (bottom), ANBP-B at 3.59 min (H) MS^E^ spectrum of ANBP-A in the control at day 3. No ANBP-B was observed in in the *Paucibacter toxinivorans* 2C20 culture at day 3, Table S1: Exponential decay rate (d^−1^) and half-life for each cyanopeptide in different condition (purified, mix, and with *M. aeruginosa* 7806 crude extract) by *Paucibacter toxinivorans* 2C20 strain. The decay rates are represented by incubation time according to the first three days, the last four days and over the total 7 days; Table S2: Chemical information of all peptides tested here, considering their chemical structure, molecular formula, molecular weight, selected reaction monitoring (SRM) transitions and retention time in UPLC/MS system.
