# Supplementary material for: Degradation of Multiple Peptides by Microcystin-Degrader Paucibacter toxinivorans (2C20)
Source: Toxins (Basel). 2021 Apr 8;13(4):265. doi: 10.3390/toxins13040265 (PMC8068134; doi:10.3390/toxins13040265)
Supplement: Supplementary file 1 [file toxins-13-00265-s001.pdf]

Supplementary Materials: Degradation of Multiple Peptides by Microcystin-Degrader *Paucibacter toxinivorans* (2C20)

Allan A. Santos, Sylvia Soldatou, Valeria Freitas de Magalhães, Sandra M. F. O. Azevedo, Dolores Camacho-Muñoz, Linda A. Lawton and Christine Edwards

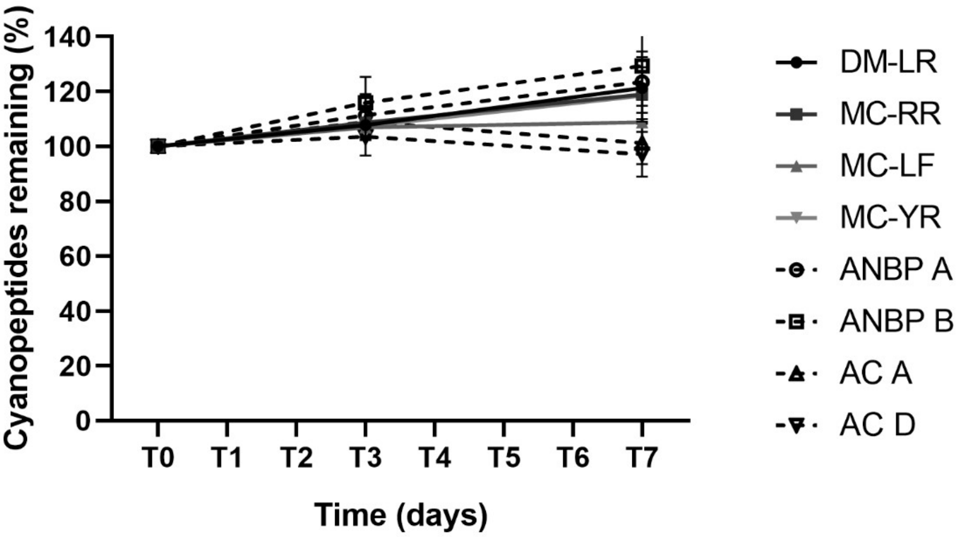

Figure S1. Negative control without *Paucibacter toxinivorans* culture for cyanopeptides degradation into mix condition. The data are expressed in percentage of remained concentration of peptides considering mean and standard deviation (n = 3).

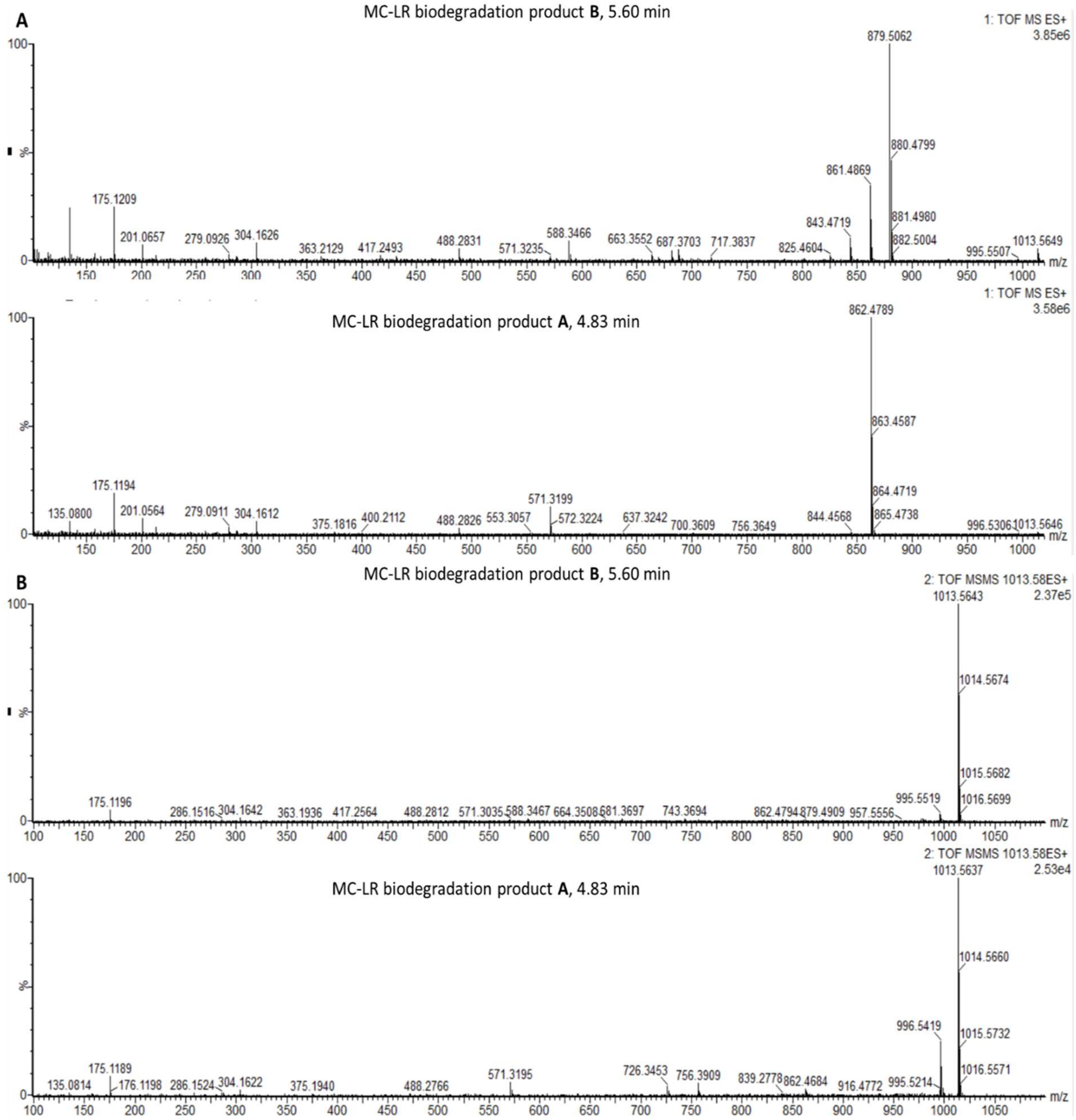

Figure S2. (**A**) Bottom: MS^E spectrum of biodegradation product A at 4.83 min identified in the *Paucibacter toxinivorans* 2C20 culture sample in pure MC-LR conditions at day 7, Top: MS^E spectrum of biodegradation product B at 5.60 min identified in the *Paucibacter toxinivorans* 2C20 culture sample in in pure MC-LR conditions at day 7. (**B**) Bottom: MS/MS spectrum of *m/z* 862.4789, Top: MS/MS spectrum of *m/z* 879.5062.

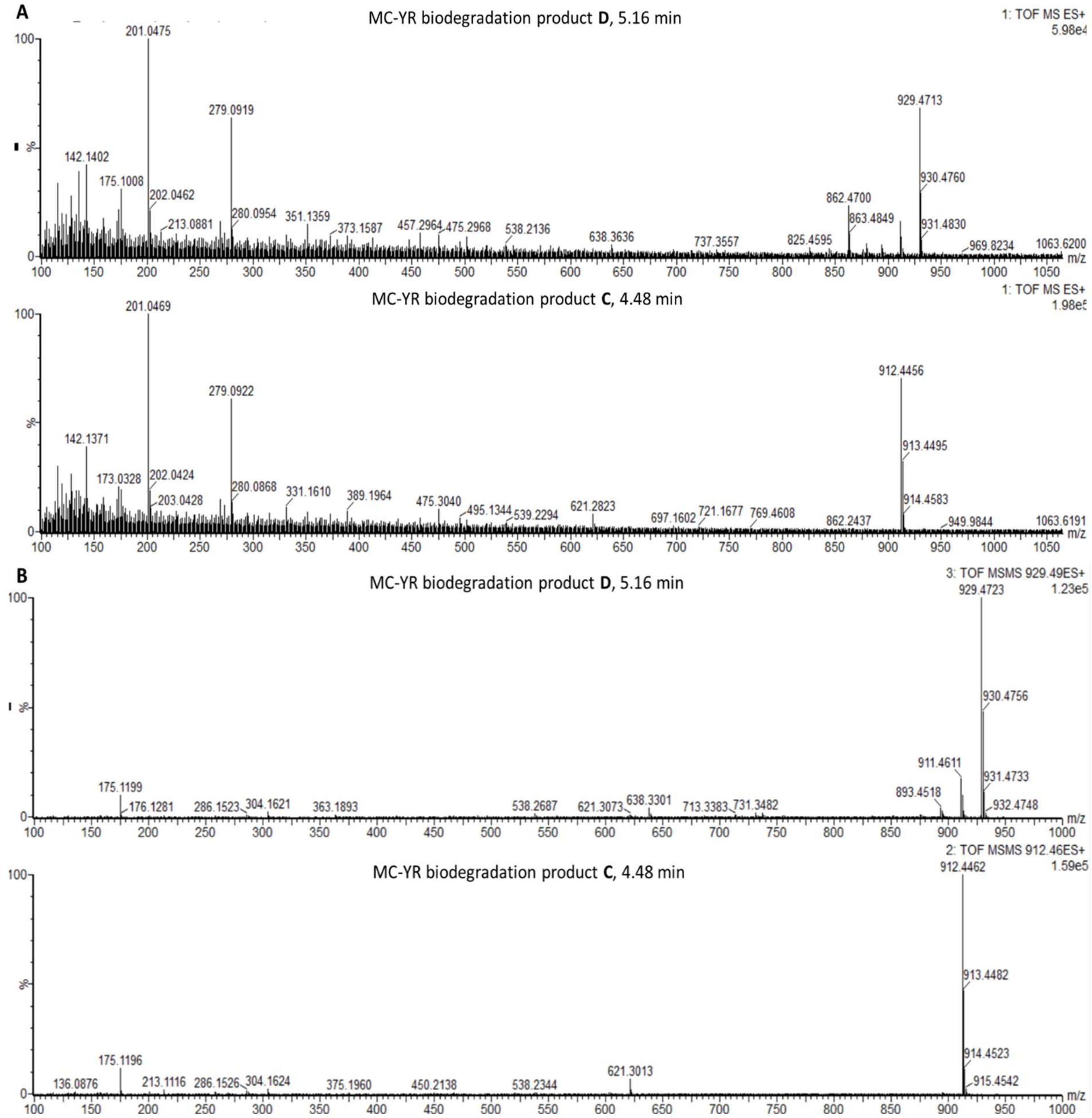

Figure S3. (**A**) Bottom: MS^E spectrum of biodegradation product C at 4.48 min identified in the *Paucibacter toxinivorans* 2C20 culture sample in peptide mixture condition at day 7, Top: MS^E spectrum of biodegradation product C at 5.16 min identified in the *Paucibacter toxinivorans* 2C20 culture sample in peptide mixture condition at day 7. (**B**) Bottom: MS/MS spectrum of *m*/*z* 912.4462, Top: MS/MS spectrum of *m*/*z* 929.4723.

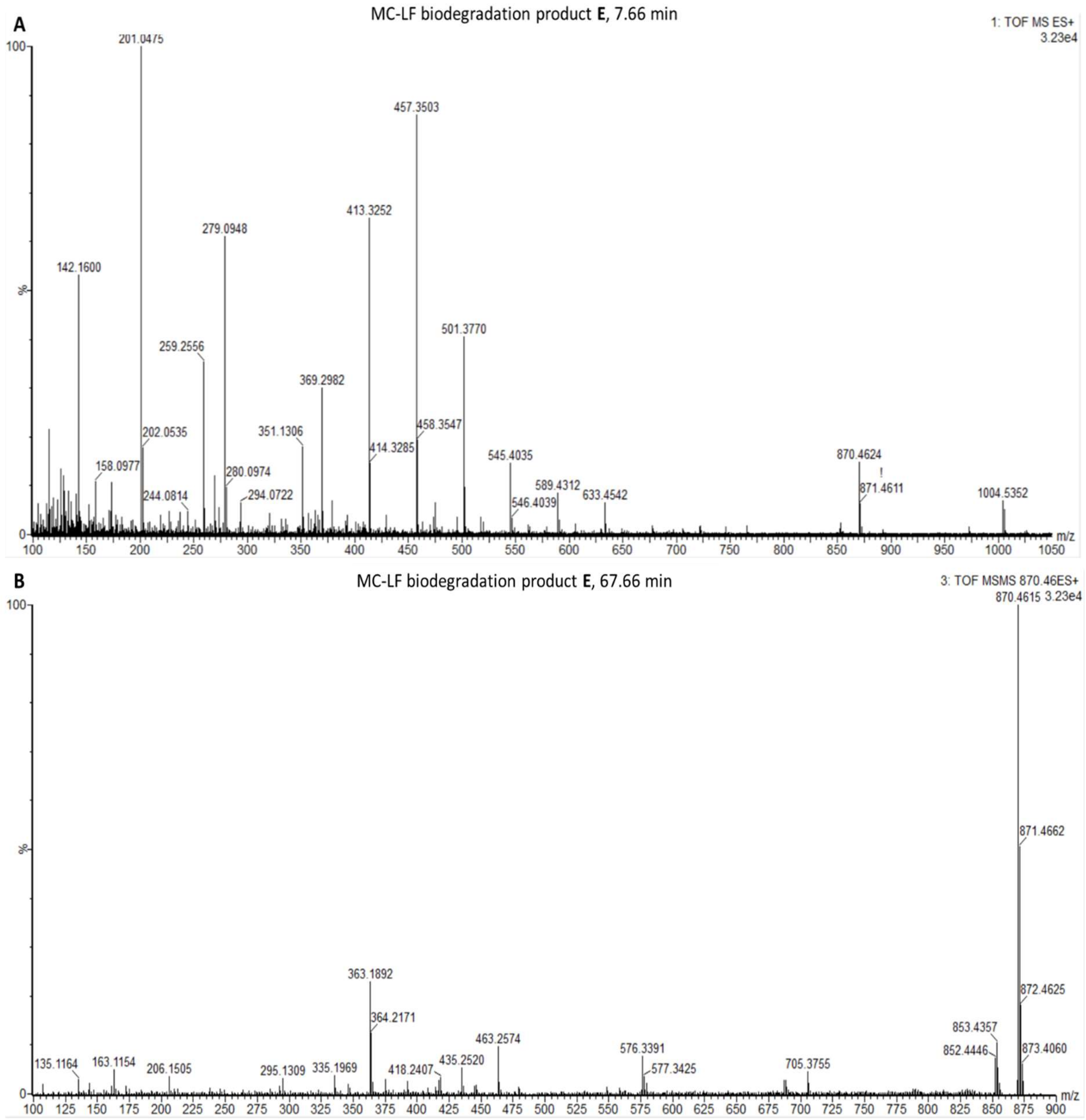

Figure S4. (**A**) MS^E spectrum of biodegradation product E at 7.66 min identified in the *Paucibacter toxinivorans* 2C20 culture sample in peptide mixture condition at day 7, (**B**) MS/MS spectrum of *m*/*z* 870.4624.

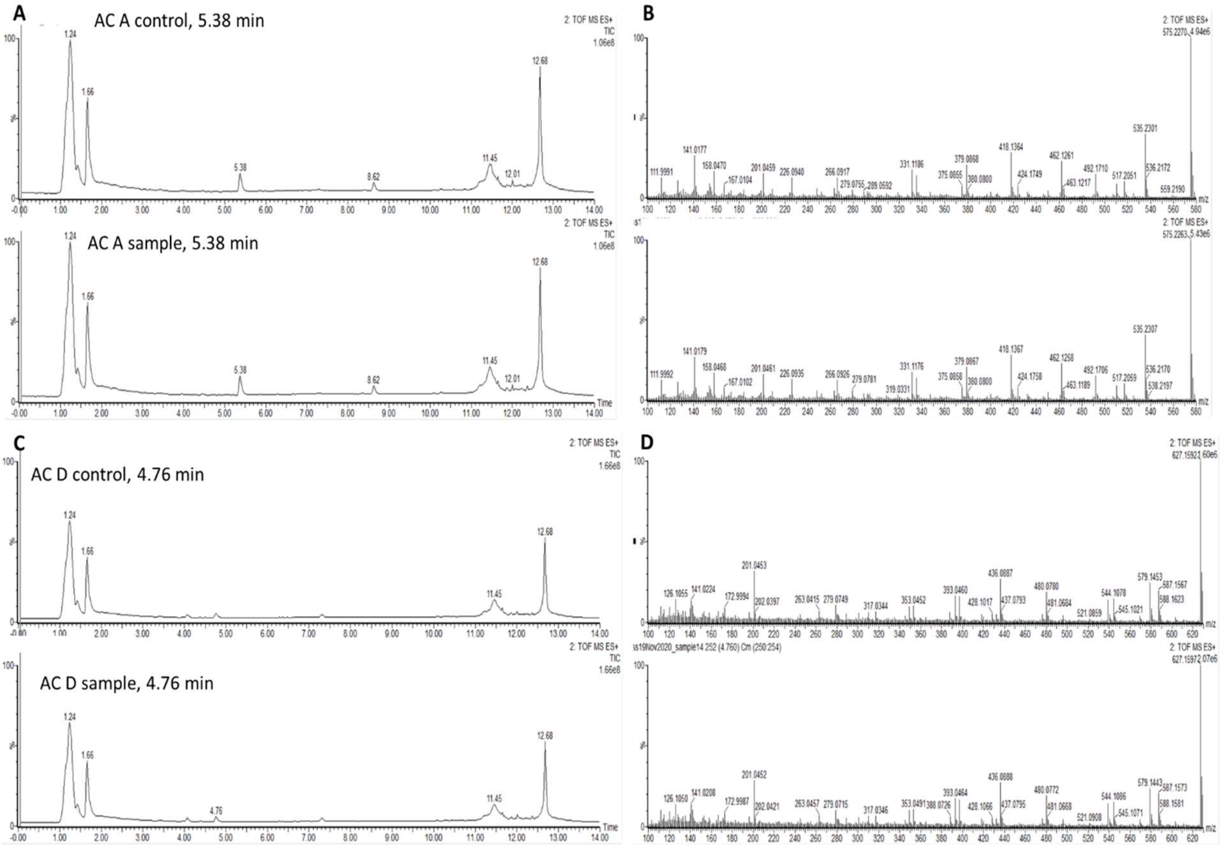
A
AC A control, 5.38 min
AC A sample, 5.38 min
B
C
AC D control, 4.76 min
AC D sample, 4.76 min
D
2: TOF MS ES+
TIC

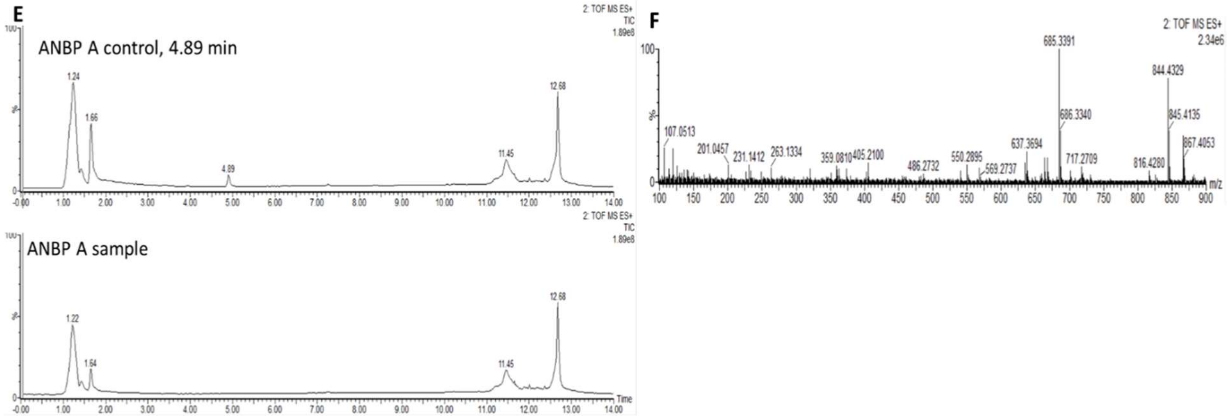
E
ANBP A control, 4.89 min
ANBP A sample
F
2: TOF MS ES+
685.3391
844.4329

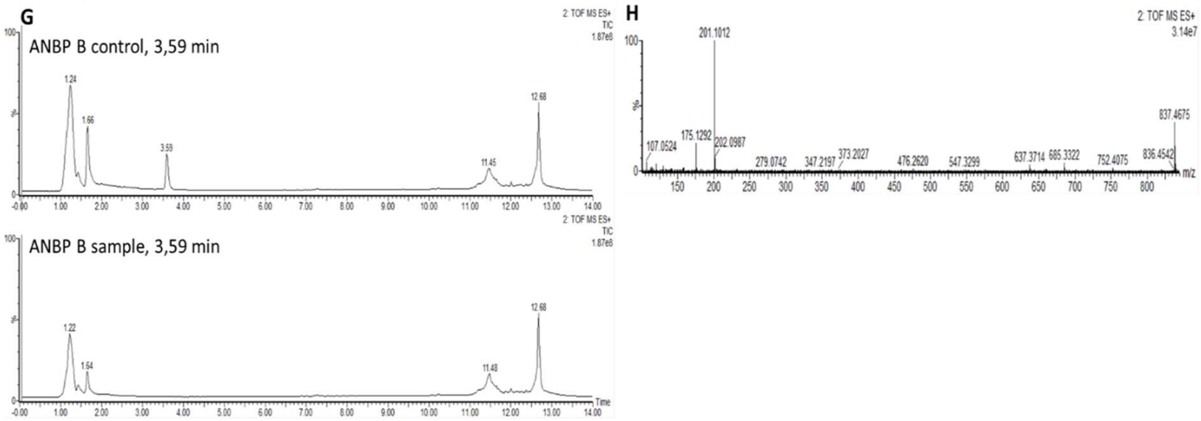

Figure S5. (**A**) UPLC chromatogram of the control (top) and of the *Paucibacter toxinivorans* 2C20 culture at day 3 (bottom), AC-A at 5.38 min (**B**) MS^E spectrum of AC-A in the control (top) and in the *Paucibacter toxinivorans* 2C20 culture at day 3 (bottom). (**C**) UPLC chromatogram of the control (top) and of the *Paucibacter toxinivorans* 2C20 culture at day 3 (bottom), AC-D at 4.76 min (**D**) MS^E spectrum of AC-D in the control (top) and in the *Paucibacter toxinivorans* 2C20 culture at day 3 (bottom). (**E**) UPLC chromatogram of the control (top) and of the *Paucibacter toxinivorans* 2C20 culture at day 3 (bottom), ANBP-A at 4.89 min (**F**) MS^E spectrum of ANBP-A in the control at day 3. No ANBP-A was observed in in the *Paucibacter toxinivorans* 2C20 culture at day 3. (**G**) UPLC chromatogram of the control (top) and of the *Paucibacter toxinivorans* 2C20 culture at day 3 (bottom), ANBP-B at 3.59 min (**H**) MS^E spectrum of ANBP-A in the control at day 3. No ANBP-B was observed in in the *Paucibacter toxinivorans* 2C20 culture at day 3.

Table S1. Exponential decay rate (d^{-1}) and half-life for each cyanopeptide in different condition (purified, mix, and with *M. aeruginosa* 7806 crude extract) by *Paucibacter toxinivorans* 2C20 strain. The decay rates are represented by incubation time according to the first three days, the last four days and over the total 7 days.

	Decay rate of cyanopeptides			Half-Life ($T_{1/2}$)
	Bi-phasic step		Over 7 days	
	First 3 days	Last 4 days		
**Purified cyanopeptide**				
MC-LR	0.109 ± 0.01	0.394 ± 0.02	0.272 ± 0.01	4.5
DM-LR	0.203 ± 0.01	0.719 ± 0.01	0.498 ± 0.01	3.5
MC-RR	0.240 ± 0.04	0.435 ± 0.09	0.351 ± 0.07	3.0
MC-LF	0.233 ± 0.02	0.478 ± 0.01	0.372 ± 0.01	3.5
MC-YR	0.177 ± 0.02	0.819 ± 0.09	0.544 ± 0.44	3.5
ANBP-A	2.363 ± 0.01	**	**	1.5
ANBP-B	2.350 ± 0.04	**	**	1.5
AC-A	1.643 ± 0.01	**	**	1.5
AC-D	2.607 ± 0.04	**	**	1.5
**Cyanopeptides (mix)**				
MC-LR + peptide mix	0.09 ± 0.05	0.215 ± 0.04	0.162 ± 0.01	5.5
DM-LR in mix	0.088 ± 0.02	0.209 ± 0.01	0.157 ± 0.01	5.5
MC-RR in mix	0.068 ± 0.02	0.100 ± 0.01	0.086 ± 0.01	7.0
MC-LF in mix	0.022 ± 0.02	0.077 ± 0.01	0.054 ± 0.01	>7.0
MC-YR in mix	0.110 ± 0.02	0.236 ± 0.02	0.182 ± 0.01	5.0
ANBP-A in mix	*	*	*	*
ANBP-B in mix	*	*	*	*
AC-A in mix	*	*	*	*

AC-D in mix	*	*	*	*
**MC-LR + *M.aeruginosa* 7806 crude extract**	2.33 ± 0.01	**	**	1.5

*respective cyanopeptide was not detected at any time even time 0, which makes an unattainable decay rate calculation. **respective cyanopeptide was completely degraded over 3rd day, so there was no an exactly decay rate for this interval.

Table S2. Chemical information of all peptides tested here, considering their chemical structure, molecular formula, molecular weight, selected reaction monitoring (SRM) transitions and retention time in UPLC/MS system.

Compounds	Chemical Structure	Molecular Formula	Molecular Weight (g/mol)	MRM Transitions ([M+H]* or [M+2H]**)	Retention Time
Microcystin-LR (MC-LR)	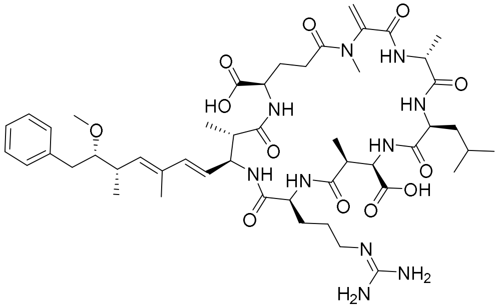	$C_{49}H_{74}N_{10}O_{12}$	995.2	995.6>135.239*	2.78
[Asp3]Microcystin-LR (DM-LR)	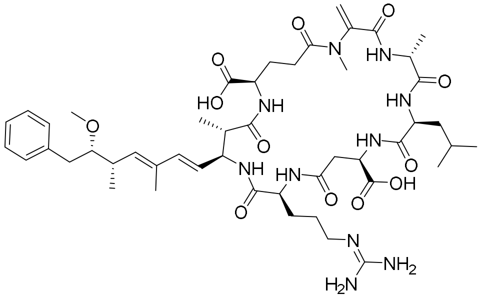	$C_{48}H_{72}N_{10}O_{12}$	981.1	981.596>135.239*	2.81
Microcystin-RR (MC-RR)	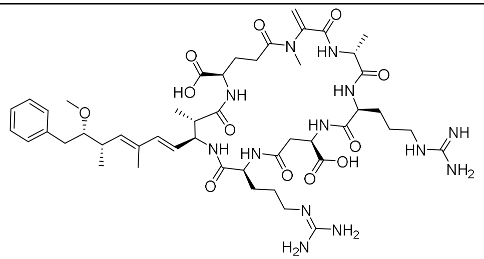	$C_{49}H_{75}N_{13}O_{12}$	1038.2	520>135.24**	1.83

Microcystin-LF (MC-LF)	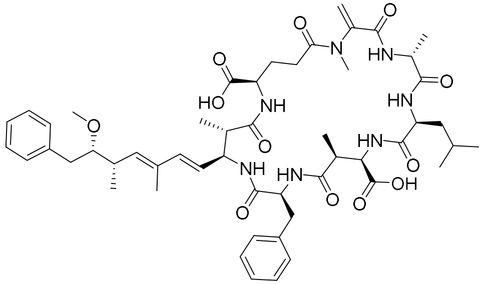	$C_{52}H_{71}N_7O_{12}$	986.2	986.596>135.245*	4.40
Microcystin-YR (MC-YR)	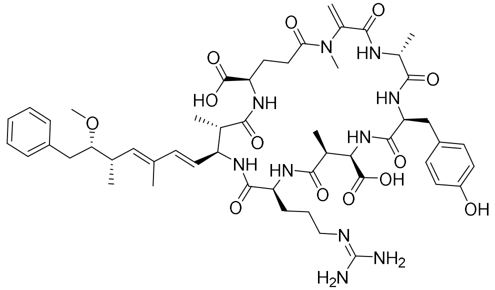	$C_{52}H_{72}N_{10}O_{13}$	1045.2	1045.568>135.303*	2.57
Anabaenopeptin A (ANPB-A)	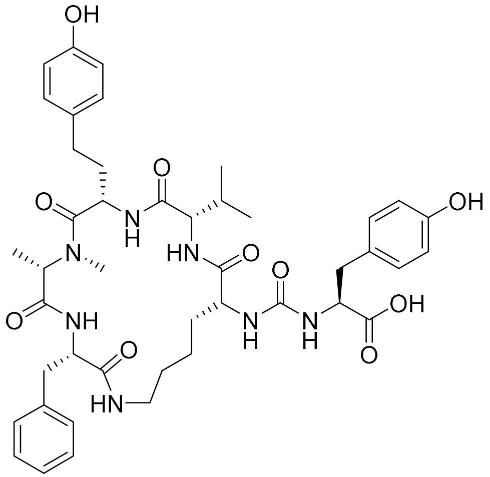	$C_{44}H_{57}N_7O_{10}$	843.9	844.48>83.96*	1.85

Anabaenopeptin B (ANBP-B)	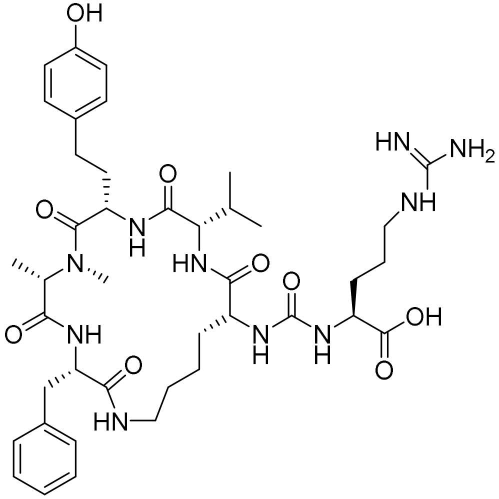	$C_{41}H_{60}N_{10}O_9$	837	837.521>201.021*	1.56
Aerucyclamide A (AC-A)	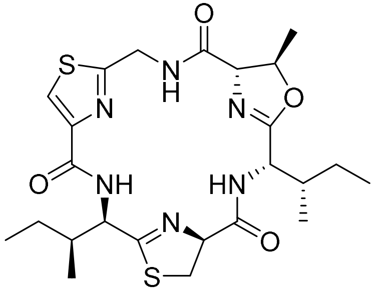	$C_{24}H_{34}N_6O_4S_2$	534.7	535..287>140.907*	2.58
Aerucyclamide D (AC-D)	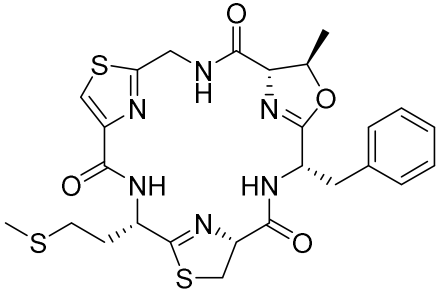	$C_{26}H_{30}N_6O_4S_3$	586.8	587.13>539.125*	2.32

Cyclosporine A (CYCL)	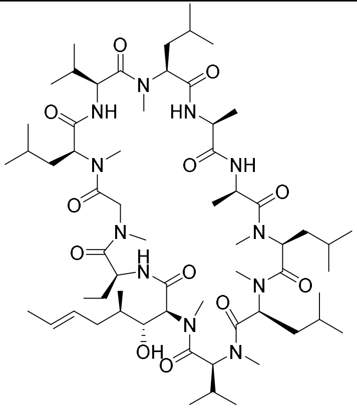	$C_{62}H_{1}11N_{11}O_{12}$	1202.6	1224.841>1112.809*	3.57
[Glu1]Fibrinopeptide-B (FIB)	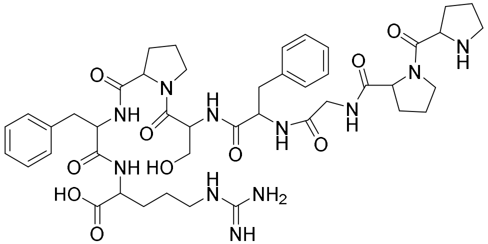	$C_{66}H_{95}N_{19}O_{26}$	1570.6	786.075>119.95**	1.25
Leucine-Enkephalin (LEU-ENK)	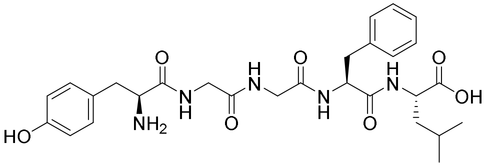	$C_{28}H_{37}N_{5}O_{7}$	555.6	556.313>119.951*	1.43

Oxytocin (OXYT)	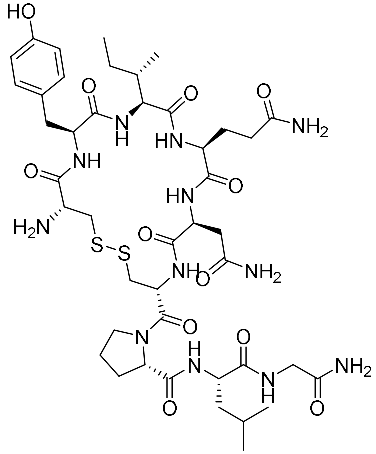	$C_{43}H_{66}N_{12}O_{12}S_2$	1007.2	1007.458>723.307*	1.30

*dominant ion observed in mass spectrometry analysis when singly protonated or **doubly protonated ion.
